# Supplementary material for: Polyketide synthase gene clusters in the ericoid mycorrhizal fungus Oidiodendron maius: genome-wide prediction, expression profiling, and functional characterization of knockout mutants
Source: Front Plant Sci. 2026 Sep 16;17:1924430. doi: 10.3389/fpls.2026.1924430 (PMC13623903; doi:10.3389/fpls.2026.1924430)
Supplement: Supplementary file 1 [file SupplementaryFile1.zip › Supplementary Materials/Supplementary Folder 1.zip!/Oidma1_AssemblyScaffolds_Repeatmasked/knownclusterblast/cluster14/jgi.p_Oidma1_102294_mibig_hits.html]

| MIBiG Protein | Description | MIBiG Cluster | MiBiG Product | % ID | % Coverage | BLAST Score | E-value |
| --- | --- | --- | --- | --- | --- | --- | --- |
| ABZ01830.1 | ESC\_reductase | BGC0001272 | Elsinochrome\_B\_/\_elsinochrome\_C\_biosynthetic\_gene\_cluster | 50 | 92.2535211268 | 261 | 1.3e-69 |
| AAC49205.1 | putative\_ketoreductase | BGC0000152 | Sterigmatocystin\_biosynthetic\_gene\_cluster | 50 | 89.7887323944 | 253 | 2.1e-67 |
| ACI31322.1 | AflM | BGC0000011 | Aflatoxin/sterigmatocystin\_biosynthetic\_gene\_cluster | 50 | 90.1408450704 | 252 | 6.1e-67 |
| CBF90105.1 | oxidoreductase,\_putative\_(Eurofung) | BGC0001271 | Emericellin\_biosynthetic\_gene\_cluster | 50 | 89.7887323944 | 251 | 1.4e-66 |
| AAS90099.1 | Ver-1 | BGC0000006 | Aflatoxin\_biosynthetic\_gene\_cluster | 49 | 89.7887323944 | 250 | 2.3e-66 |
| AAS90008.1 | Ver-1 | BGC0000007 | Aflatoxin\_biosynthetic\_gene\_cluster | 49 | 89.7887323944 | 250 | 2.3e-66 |
| AAS90030.1 | Ver-1 | BGC0000008 | Aflatoxin\_biosynthetic\_gene\_cluster | 49 | 89.7887323944 | 250 | 2.3e-66 |
| AAS90056.1 | Ver-1 | BGC0000009 | Aflatoxin\_biosynthetic\_gene\_cluster | 49 | 89.7887323944 | 250 | 2.3e-66 |
| AAS90076.1 | Ver-1 | BGC0000010 | Aflatoxin\_biosynthetic\_gene\_cluster | 49 | 89.7887323944 | 250 | 2.3e-66 |
| BAE71323.1 | reductase/dehydrogenase | BGC0000004 | Aflatoxin\_biosynthetic\_gene\_cluster | 49 | 89.7887323944 | 249 | 5.1e-66 |
| AFO85456.1 | reductase/oxidase | BGC0000391 | Methylpendolmycin/pendolmycin\_biosynthetic\_gene\_cluster | 40 | 89.4366197183 | 175 | 9.4e-44 |
| AGN74904.1 | ketoreductase | BGC0000459 | Griseoviridin\_/\_viridogrisein\_biosynthetic\_gene\_cluster | 40 | 91.5492957746 | 165 | 9.7e-41 |
| AGH68923.1 | oxidoreductase | BGC0001083 | Merochlorin\_biosynthetic\_gene\_cluster | 40 | 91.9014084507 | 165 | 1.3e-40 |
| AEF33074.1 | 3-oxoacyl-[acyl-carrier\_protein]\_reductase | BGC0001039 | Pyridomycin\_biosynthetic\_gene\_cluster | 38 | 91.9014084507 | 153 | 3.8e-37 |
| ADI71448.1 | putative\_oxygenase-reductase | BGC0000203 | BE-7585A\_biosynthetic\_gene\_cluster | 36 | 100.0 | 153 | 5e-37 |
| AHH25583.1 | 1-cyclohexenylcarbonyl\_CoA\_reductase | BGC0000957 | Ansatrienin\_(mycotrienin)\_biosynthetic\_gene\_cluster | 34 | 88.3802816901 | 152 | 6.5e-37 |
| AAT12286.1 | LtxD | BGC0000384 | Lyngbyatoxin\_biosynthetic\_gene\_cluster | 36 | 90.1408450704 | 149 | 7.2e-36 |
| WP\_030267262.1 | 3-oxoacyl-ACP\_reductase | BGC0001308\_c2 | Simocyclinone\_D9\_/\_simocyclinone\_D10\_/\_simocyclinone\_D11 | 36 | 89.7887323944 | 148 | 9.4e-36 |
| CAH10112.1 | putative\_oxidase-reducatse | BGC0000268 | Sch47554\_/\_Sch47555\_biosynthetic\_gene\_cluster | 34 | 94.014084507 | 147 | 2.7e-35 |
| ACP19359.1 | SaqN | BGC0000267 | Saquayamycin\_Z\_/\_galtamycin\_B\_biosynthetic\_gene\_cluster | 35 | 92.9577464789 | 146 | 3.6e-35 |
| AAP13848.2 | C5-ketoreductase | BGC0000093 | Meilingmycin\_biosynthetic\_gene\_cluster | 37 | 95.7746478873 | 146 | 3.6e-35 |
| ADB02849.1 | AzicF | BGC0000202 | Azicemicin\_biosynthetic\_gene\_cluster | 37 | 90.8450704225 | 146 | 4.7e-35 |
| AAL15603.1 | SimJ1 | BGC0000270 | Simocyclinone\_biosynthetic\_gene\_cluster | 37 | 89.7887323944 | 146 | 4.7e-35 |
| AAG34187.1 | SimJ1 | BGC0000835 | Simocyclinone\_biosynthetic\_gene\_cluster | 37 | 89.7887323944 | 146 | 4.7e-35 |
| AAK06807.1 | putative\_3-keto-acyl-reductase\_SimD2 | BGC0001072 | Simocyclinone\_biosynthetic\_gene\_cluster | 37 | 89.7887323944 | 146 | 4.7e-35 |
| BAV17004.1 | putative\_oxygenase-reductase | BGC0001384 | Saprolmycin\_biosynthetic\_gene\_cluster | 35 | 93.661971831 | 144 | 1.4e-34 |
| CAC37883.1 | putative\_3-oxoacyl-[acyl-carrier\_protein]\_reductase | BGC0000038 | Coelimycin\_biosynthetic\_gene\_cluster | 34 | 92.2535211268 | 144 | 2.3e-34 |
| AAX57196.2 | auricin\_polyketide\_oxygenase-reductase | BGC0000201 | Auricin\_biosynthetic\_gene\_cluster | 33 | 100.352112676 | 143 | 4e-34 |
| AAL15586.1 | Sim8 | BGC0000270 | Simocyclinone\_biosynthetic\_gene\_cluster | 33 | 92.2535211268 | 142 | 8.8e-34 |
| AAK06790.1 | putative\_reductase\_SimA9 | BGC0001072 | Simocyclinone\_biosynthetic\_gene\_cluster | 33 | 92.2535211268 | 142 | 8.8e-34 |
| EHM27510.1 | monooxygenase\_FAD-binding | BGC0000235 | Kiamycin\_biosynthetic\_gene\_cluster | 35 | 102.112676056 | 141 | 2e-33 |
| WP\_030269276.1 | short-chain\_dehydrogenase | BGC0001308 | Simocyclinone\_D9\_/\_simocyclinone\_D10\_/\_simocyclinone\_D11 | 33 | 92.2535211268 | 138 | 1.3e-32 |
| AAF00206.1 | oxygenase-reductase | BGC0000276\_c2 | Urdamycin\_biosynthetic\_gene\_cluster | 33 | 100.0 | 134 | 1.8e-31 |
| AGO50615.1 | oxygenase-reductase | BGC0000229 | Grincamycin\_biosynthetic\_gene\_cluster | 33 | 100.352112676 | 133 | 3.1e-31 |
| ACU71635.1 | short-chain\_dehydrogenase/reductase\_SDR | BGC0001154 | Cacibiocin\_biosynthetic\_gene\_cluster | 36 | 89.7887323944 | 132 | 7e-31 |
| ACM68692.1 | putative\_oxidoreductase | BGC0000298 | Aeruginosin\_biosynthetic\_gene\_cluster | 32 | 91.5492957746 | 129 | 7.7e-30 |
| ABX71123.1 | Lct40 | BGC0000238 | Lactonamycin\_biosynthetic\_gene\_cluster | 32 | 90.1408450704 | 129 | 7.7e-30 |
| AAY93361.1 | glucose\_1-dehydrogenase | BGC0000413 | Pyoverdine\_biosynthetic\_gene\_cluster | 33 | 93.3098591549 | 128 | 1e-29 |
| AAS79466.1 | putative\_post-PKS\_ketoreductase | BGC0000035 | Chalcomycin\_biosynthetic\_gene\_cluster | 35 | 89.4366197183 | 124 | 1.5e-28 |
| ABB52548.1 | 3-oxoacyl-(acyl-carrier-protein)-reductase | BGC0000047 | Dihydrochalcomycin\_biosynthetic\_gene\_cluster | 34 | 89.4366197183 | 124 | 1.5e-28 |
| AHL46721.1 | ketoreductase | BGC0001179 | Rishirilide\_B\_biosynthetic\_gene\_cluster | 32 | 90.1408450704 | 124 | 1.9e-28 |
| ABD29269.1 | 3-ketoacyl-acyl\_carrier\_protein\_reductase,\_putative | BGC0000943 | Staphylobactin\_biosynthetic\_gene\_cluster | 33 | 90.4929577465 | 123 | 3.3e-28 |
| ANC94959.1 | AlmK | BGC0001396 | Aldgamycin\_biosynthetic\_gene\_cluster | 34 | 89.4366197183 | 123 | 3.3e-28 |
| ADD83002.1 | PtnO1 | BGC0001156 | Platencin\_biosynthetic\_gene\_cluster | 34 | 91.5492957746 | 120 | 2.8e-27 |
| ADB23430.1 | putative\_short\_chain\_dehydrogenase | BGC0000155 | T-toxin\_biosynthetic\_gene\_cluster | 34 | 77.4647887324 | 119 | 4.7e-27 |
| CBW45710.1 | putative\_ketoreductase | BGC0000952\_c3 | Pristinamycin\_biosynthetic\_gene\_cluster | 35 | 91.1971830986 | 119 | 6.1e-27 |
| ACO31265.1 | Ptm01 | BGC0001140 | Platensimycin\_/\_platencin\_biosynthetic\_gene\_cluster | 33 | 91.5492957746 | 118 | 1e-26 |
| ADD83006.1 | PtnO8 | BGC0001156 | Platencin\_biosynthetic\_gene\_cluster | 33 | 95.4225352113 | 117 | 2.3e-26 |
| ACU59350.1 | short-chain\_dehydrogenase/reductase\_SDR | BGC0000839 | Flexirubin\_biosynthetic\_gene\_cluster | 30 | 89.7887323944 | 117 | 3e-26 |
| ACO31295.1 | PtmO8 | BGC0001140 | Platensimycin\_/\_platencin\_biosynthetic\_gene\_cluster | 33 | 93.661971831 | 116 | 5.2e-26 |
| ADC45556.1 | 2-hydroxycyclohexanecarboxyl-CoA\_dehyrogenase | BGC0000093 | Meilingmycin\_biosynthetic\_gene\_cluster | 32 | 98.5915492958 | 114 | 2e-25 |
| AFY23045.1 | TamN | BGC0000274 | Tetarimycin\_biosynthetic\_gene\_cluster | 31 | 93.3098591549 | 113 | 4.4e-25 |
| AIL50168.1 | putative\_ketoreductase | BGC0000213 | Colabomycin\_biosynthetic\_gene\_cluster | 29 | 88.3802816901 | 111 | 1.7e-24 |
| CAK50780.1 | ketoreductase | BGC0000247 | Mithramycin\_biosynthetic\_gene\_cluster | 32 | 91.9014084507 | 109 | 4.9e-24 |
| CAA07765.1 | ketoreductase | BGC0000246 | Mithramycin\_biosynthetic\_gene\_cluster | 32 | 91.9014084507 | 109 | 4.9e-24 |
| AAZ55906.1 | 2,3-dihydro-2,3-dihydroxybenzoate\_dehydrogenase;\_RBL00455 | BGC0000359 | Fuscachelin\_biosynthetic\_gene\_cluster | 31 | 92.6056338028 | 108 | 1.4e-23 |
| AAZ23052.1 | probable\_dehydrogenase | BGC0000291 | A54145\_biosynthetic\_gene\_cluster | 31 | 91.9014084507 | 108 | 1.8e-23 |
| ANY57964.1 | MtcE\_3-ketoacyl-ACP\_reductase | BGC0001369 | Metatricycloene\_biosynthetic\_gene\_cluster | 28 | 91.9014084507 | 105 | 1.2e-22 |
| ABQ04140.1 | short-chain\_dehydrogenase/reductase\_SDR | BGC0000838 | Flexirubin\_biosynthetic\_gene\_cluster | 30 | 90.1408450704 | 104 | 2e-22 |
| CAA54861.1 | ketoreductase | BGC0000231 | Griseusin\_biosynthetic\_gene\_cluster | 31 | 89.7887323944 | 103 | 4.5e-22 |
| CAJ42322.1 | ketoreductase | BGC0000273 | Steffimycin\_biosynthetic\_gene\_cluster | 26 | 98.5915492958 | 102 | 5.9e-22 |
| CAA34367.1 |  | BGC0000228 | Granaticin\_biosynthetic\_gene\_cluster | 33 | 91.1971830986 | 101 | 1.7e-21 |
| CAA09651.1 |  | BGC0000227 | Granaticin\_biosynthetic\_gene\_cluster | 33 | 91.1971830986 | 101 | 1.7e-21 |
| CAH10175.1 | ChaZ\_protein | BGC0000207 | Chartreusin\_biosynthetic\_gene\_cluster | 29 | 89.7887323944 | 99 | 8.6e-21 |
| AAB36565.1 | ketoreductase | BGC0000234 | Jadomycin\_biosynthetic\_gene\_cluster | 30 | 89.7887323944 | 98 | 1.1e-20 |
| EDY47129.1 | conserved\_hypothetical\_protein | BGC0000845 | Clavulanic\_acid\_biosynthetic\_gene\_cluster | 30 | 75.0 | 97 | 2.5e-20 |
| AAX57194.1 | auricin\_polyketide\_ketoreductase | BGC0000201 | Auricin\_biosynthetic\_gene\_cluster | 31 | 89.7887323944 | 96 | 5.6e-20 |
| AHD25940.1 | ChdT | BGC0000208 | Chelocardin\_biosynthetic\_gene\_cluster | 29 | 89.7887323944 | 95 | 9.5e-20 |
| ADD83005.1 | PtnO7 | BGC0001156 | Platencin\_biosynthetic\_gene\_cluster | 34 | 70.4225352113 | 92 | 6.1e-19 |
| ACO31293.1 | PtmO7 | BGC0001140 | Platensimycin\_/\_platencin\_biosynthetic\_gene\_cluster | 33 | 70.4225352113 | 92 | 1e-18 |
| ABI75108.1 | short-chain\_alcohol\_dehydrogenase | BGC0000928 | T3\_toxin\_biosynthetic\_gene\_cluster | 29 | 89.0845070423 | 90 | 3.1e-18 |
| ABI75134.1 | short-chain\_alcohol\_dehydrogenase | BGC0000887 | AWQC131C\_biosynthetic\_gene\_cluster | 29 | 89.4366197183 | 89 | 6.8e-18 |
| AAN85505.1 | short-chain\_dehydrogenase/reductase | BGC0001101 | Leinamycin\_biosynthetic\_gene\_cluster | 27 | 96.4788732394 | 82 | 6.4e-16 |
| BAU98027.1 | dehydrogenase | BGC0001386 | Isofuranonaphthoquinone\_biosynthetic\_gene\_cluster | 29 | 69.7183098592 | 74 | 1.7e-13 |
| ACN52304.1 | TsrN | BGC0000614 | Thiostrepton\_biosynthetic\_gene\_cluster | 32 | 74.6478873239 | 71 | 1.9e-12 |
| AAD37453.1 | putative\_ketoacyl\_reductase | BGC0000252 | Nonactin\_biosynthetic\_gene\_cluster | 24 | 69.014084507 | 55 | 1.4e-07 |
